# Risk factors for lymph node metastasis and prognosis in colorectal neuroendocrine tumours

**DOI:** 10.1007/s00384-021-04082-7

**Published:** 2022-01-08

**Authors:** Xiuli Zheng, Mingli Wu, Limian Er, Huiyan Deng, Gongning Wang, Lingyao Jin, Shengmian Li

**Affiliations:** 1 Department of Endoscopy, The Fourth Hospital of Hebei Medical University, No. 12 Jiankang Road, Chang’an District, Shijiazhuang, 050000 Hebei China; 2 Department of Pathology, The Fourth Hospital of Hebei Medical University, No. 12 Jiankang Road, Chang’an District, Shijiazhuang, 050000 Hebei China; 3 Department of Gastroenterology, the Fourth Hospital of Hebei Medical University, No. 12 Jiankang Road, Chang’an District, Shijiazhuang, 050000 Hebei China

**Keywords:** Colorectal neuroendocrine tumour, Lymph node metastasis, Prognosis

## Abstract

**Purpose:**

The detection rate of colorectal neuroendocrine tumours (CR-NETs) is increasing, but their treatment is still controversial. Lymph node metastasis is an important reference index for the selection of treatment. The aim of our study was to investigate the factors associated with lymph node metastasis and prognosis of CR-NETs.

**Methods:**

The case characteristics of patients with colorectal neuroendocrine tumours from January 2011 to December 2020 were retrospectively analysed, including age, gender, tumour size, tumour location, lymph node metastasis, pathological grade and follow-up.

**Results:**

A total of 195 cases of CR-NETs were included in this study. When 15 mm was used as the cut-off value, the sensitivity, specificity and area under the curve (AUC) of lymph node metastases were 95.9%, 95.2% and 0.986, respectively. Multivariate analysis suggested that tumour size ≥ 15 mm (OR: 30.517, 95% CI: 1.250 ~ 744.996, *p* = 0.036) and lymphovascular invasion (OR: 42.796, 95% CI: 2.882 ~ 635.571, *p* = 0.006) were independent risk factors for lymph node metastasis. Age ≥ 56 (HR: 7.434, 95% CI: 1.334 ~ 41.443, *p* = 0.022) and distant metastasis (HR: 24.487, 95% CI: 5.357 ~ 111.940, *p* < 0.001) were independent prognostic factors in multivariable analyses.

**Conclusions:**

When the size of a CR-NET is ≥ 15 mm, the risk of lymph node metastasis is higher, and it is recommended to choose the surgical method carefully. Tumour size and lymphovascular invasion were independent risk factors for lymph node metastasis. Age ≥ 56 and distant metastasis were independent prognostic factors.

## Introduction

Neuroendocrine tumours (NETs) are a kind of tumour with neuroendocrine function and malignant potential. It originates from pheochromo-like cells and has obvious heterogeneity [1]. With the use of colonoscopy, the detection rate of colorectal neuroendocrine tumours (CR-NETs) is increasing [2]. The associated risk factors are unknown; a study from Japan has shown higher levels of serum cholesterol and ferritin, metabolic syndrome and family history of cancer as factors that may explain the increasing incidence and prevalence of rectal NET [3]. Tumour sites also vary by race, with the incidence of rectal NETs in the Asian population increasing from 0.2 per 100,000 in 1973 to 0.86 per 100,000 in 2004, which is significantly higher than that in the white population [4]. At the same time, the incidence of CR-NETs is the fastest increasing among all NETs, accounting for 32.6% of all NETs and becoming the second most common NET in China [5].

It has been reported that before metastasis, the survival rate of CR-NETs is better than that of colorectal adenocarcinoma, and if metastasis occurs, the prognosis is similar to that of adenocarcinoma [6]. Standard resection with locoregional lymphadenectomy is appropriate [7]. Clearance of metastatic lymph nodes is a worthwhile objective that may contribute to long-term survival [8]. However, the choice of treatment methods for CR-NETs is still controversial at present [6, 9–12].

Similar to colorectal adenocarcinoma, lymph node metastasis is an important marker of malignancy, and the presence of lymph node metastasis is crucial to the choice of treatment. Reliable lymph node predictors are needed for clinical work [13]. Therefore, the study of lymph node metastasis and its related factors is of great significance for clinical diagnosis and treatment [14]. Studies have shown that there is a close relationship between tumour size and the risk of metastasis [10, 15–19], but it did not come up with an exact value. In this study, we examined the value of tumour size on the risk for lymph node metastasis and the factors associated with prognosis of CR-NETs.

## Methods

### Patient population

This study retrospectively analysed 195 cases of CR-NETs who were diagnosed and treated surgically at the Fourth Hospital of Hebei Medical University from January 2011 to December 2020. We established a retrospective database of patients’ medical records, including basic clinical features, pathological reports, and follow-up. The exclusion criteria were as follows: cases complicated with other malignant tumours and cases with incomplete clinical data. Our study was approved by the Ethics Committee of the Fourth Hospital of Hebei Medical University (ID: 2021KS002).

### Criteria

In the case of radical surgery, lymph node metastasis was determined by postoperative pathology. In the cases of endoscopic resection or local resection, computed tomography (CT) or magnetic resonance imaging (MRI) was used preoperatively and during follow-up to assess lymph node metastases. The diagnosis of a metastatic lymph node was based on the following criteria: (1) size criteria: the short-axis diameter of lymph nodes was greater than 8 mm for round lymph nodes and greater than 10 mm for ovoid lymph nodes; (2) morphological abnormalities: irregular contour and margin, unclear border, heterogeneous internal echoes or signal intensity [20–22].

The tumour size was assessed according to the maximum diameter of the tumour reported by pathology, and the tumour with distant metastasis was assessed according to the size of the imaging report.

Tumour stage and pathological diagnosis were defined according to the WHO Classification of Tumours of the Digestive System 5^th^ Edition (2019). Patients were classified as well-differentiated neuroendocrine tumours (NET G1: mitotic rate < 2/10 HPF and/or Ki-67 labelling index < 3%; NET G2: mitotic rate 2–20/10 HPF and/or Ki-67 labelling index 3–20%; NET G3: Ki-67 labelling index > 20%, generally < 60%), poorly differentiated neuroendocrine cancer (SCNEC and LCNEC) and mixed neuroendocrine and non-neuroendocrine neoplasms (MiNEN). The higher grade was adopted as Ki-67 labelling index, and mitotic rate figures were inconsistent.

### Analysis of tumour size and lymph node metastasis

**Fig. 1:**
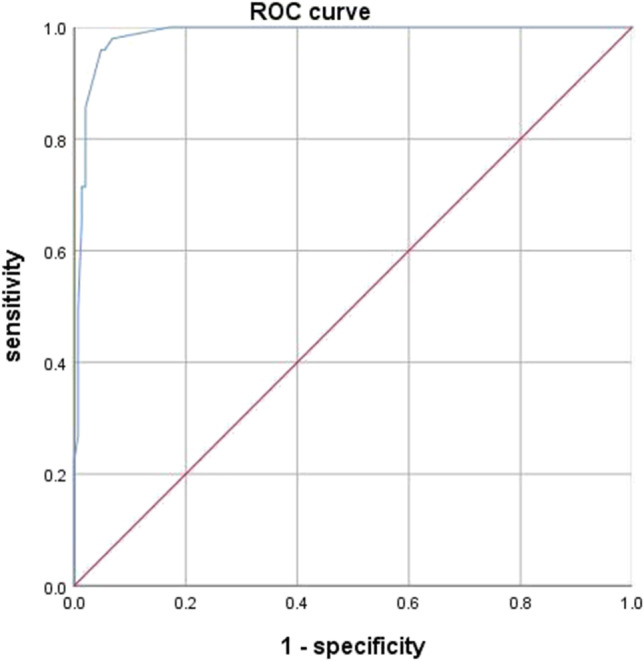
ROC curve analysis on the relationship between lymph node metastasis and tumour size. When 15 mm was used as the cut-off value, the sensitivity, specificity and area under the curve (AUC) of lymph node metastasis were 95.9%, 95.2% and 0.986, respectively

To investigate the relationship between tumour size and lymph node metastasis, a receiver operating characteristic (ROC) curve was derived. On the basis of the ROC curve, the tumour size of 15 mm was defined as an appropriate cut-off level for predicting lymph node metastasis with a high sensitivity rate of 95.9%, specificity rate of 95.2% and a area under the curve (AUC) of 0.986 (Fig. 1). Patients were stratified using a tumour size cut-off level of 15 mm to compare the risk of lymph node metastasis.

### Follow-up

Our observation outcome was NETS-related death. The last follow-up was in June 2021. Follow-up was conducted by telephone, outpatient visits or in the hospital. Failure to contact either the patient or his/her family was considered loss to follow-up.

### Statistical analysis

Continuous variables are presented as the mean with standard deviation (SD) or the median with range and were evaluated by the *t*-test. Categorical data are expressed as numbers and percentages, and analysis was conducted through a chi-square test. The predictors of lymph node metastasis were analysed by binary logistic regression. The Kaplan–Meier method was used to plot survival curves. Survival analyses were compared using Cox proportional hazard regression. Double-tailed *p* values were used for all statistical tests, and 0.05 was set as the significance threshold. Statistical analysis was performed using IBM SPSS Statistics v.25.0.0 (IBM Corp, New York). The survival curves were produced by GraphPad Prism 8.0.1 (GraphPad Software Inc., San Diego).

## Results

**Fig. 2:**
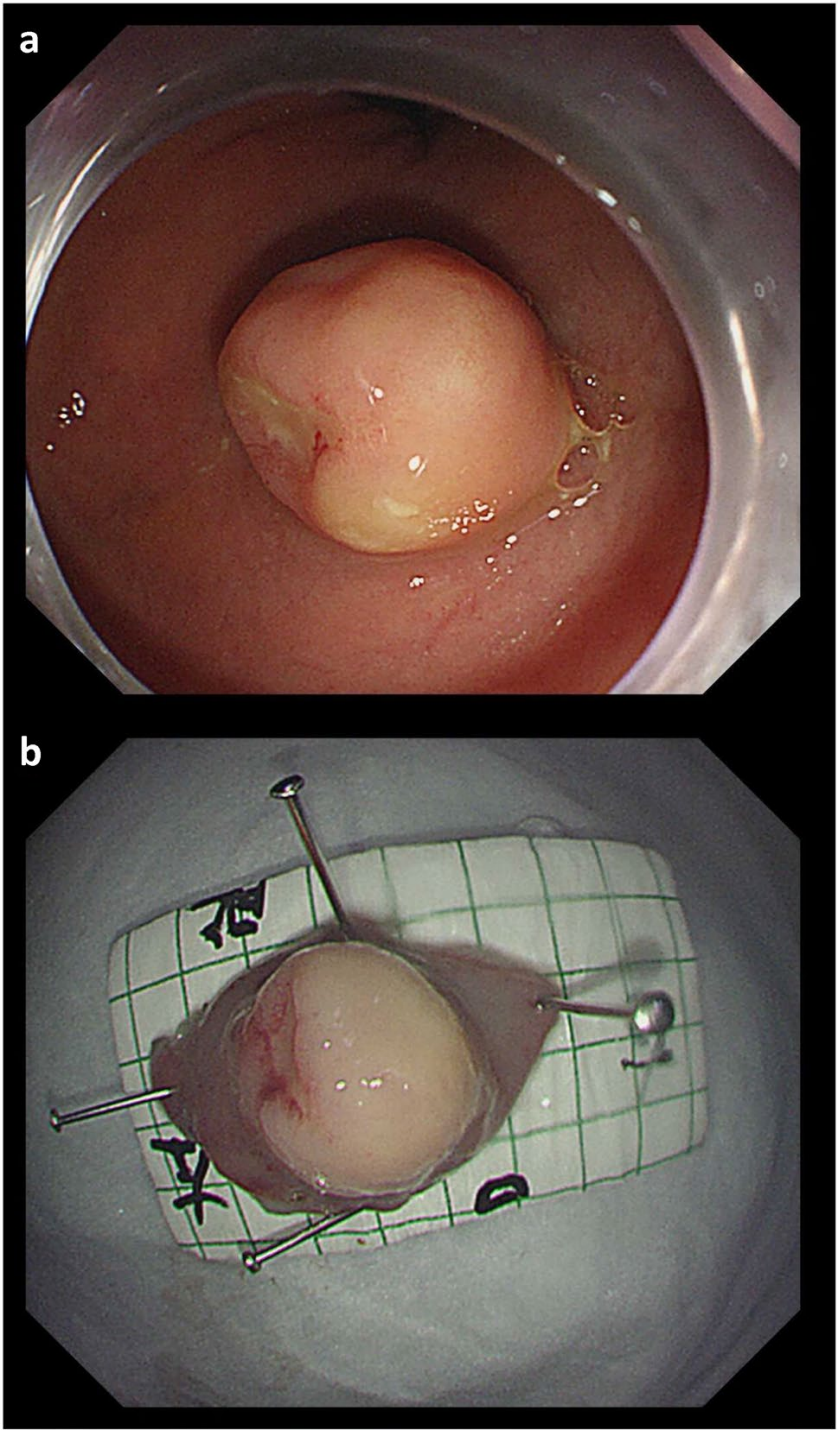
A NET G1, 12 mm in size, without lymph node metastasis. **a** Endoscopic feature of a tumour with central depression in the lower rectum. **b** The tumour was excised by ESD, and the postoperative specimen was fixed on a calibrated foam plate

**Fig. 3:**
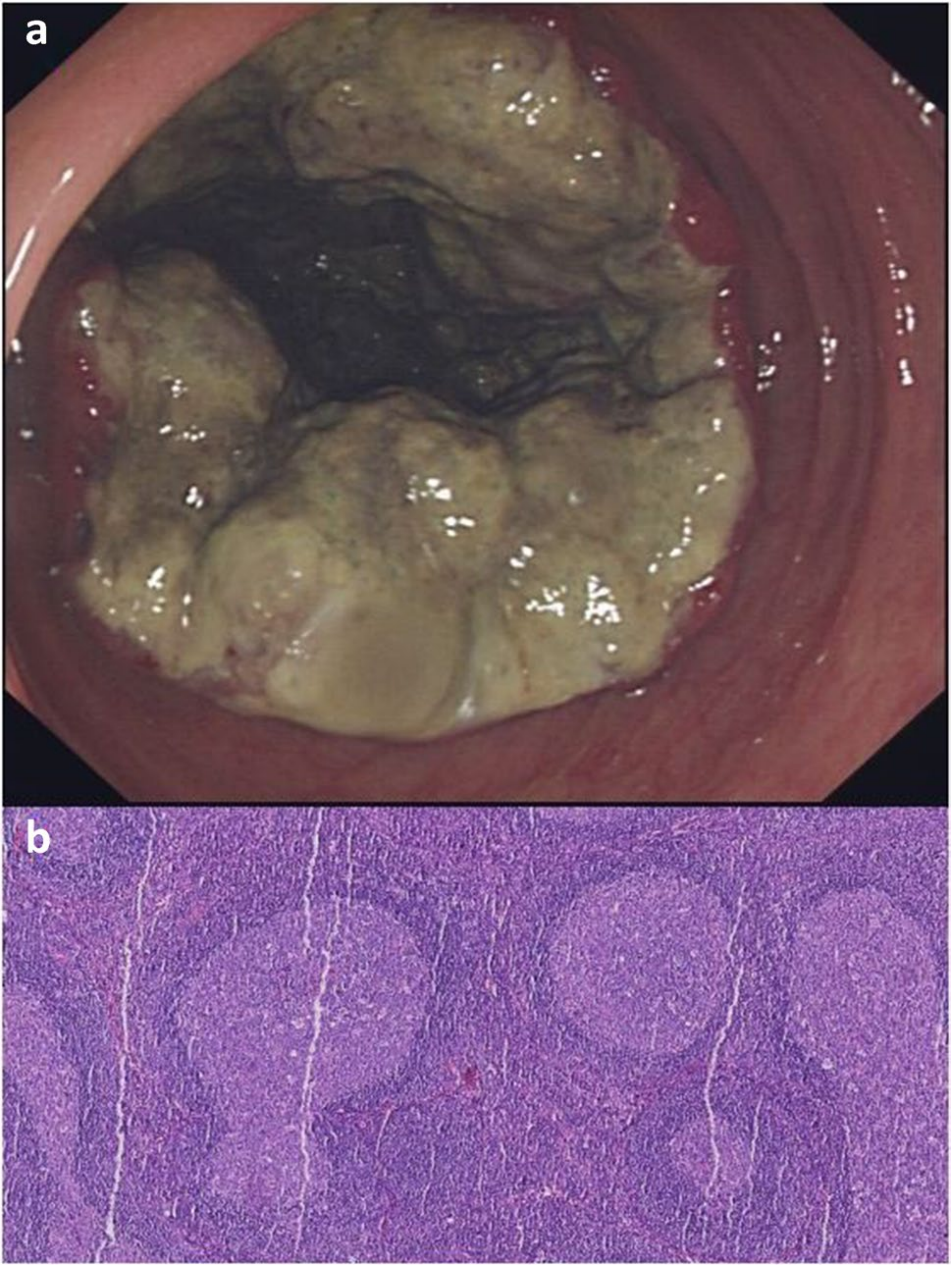
A NEC, 60 mm in size, Ki-67 index was 80%. **a** Protuberant lesions in the transverse colon. **b** Lymph node was negative

**Fig. 4:**
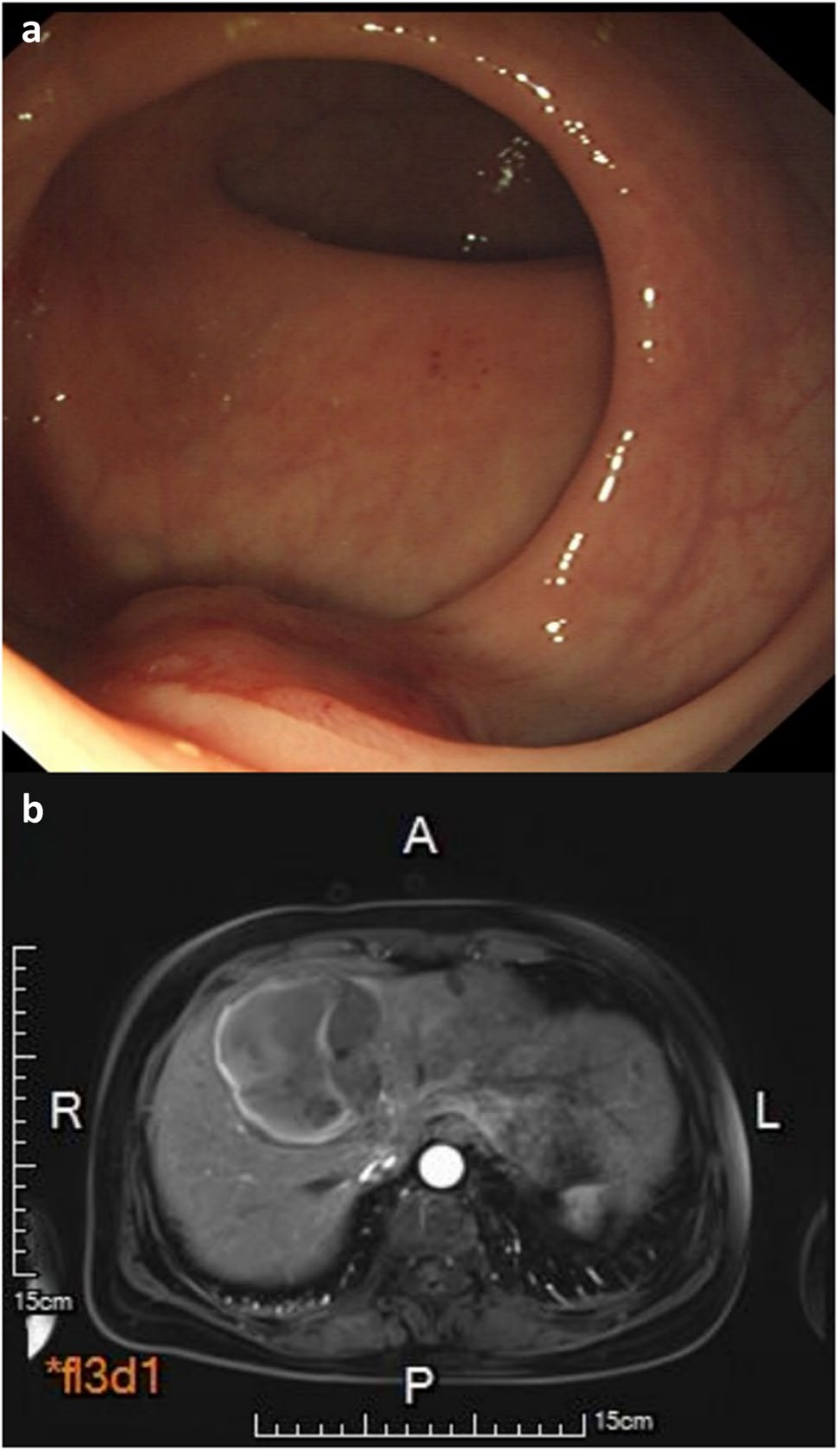
A NET G2, 20 mm in size. **a** Propelled lesion on the left wall about 3–5 cm from the anus, slightly hyperaemic on the surface mucosa. **b** MRI indicated multiple liver metastases

A total of 195 cases of CR-NETs were included in this study, including 31 cases of colon tumour and 164 cases of rectum tumour. The male-to-female ratio was 120:75, the median age was 56 years and the tumour size was 15.52 ± 17.41 mm. Among them, 111 patients underwent endoscopic resection (Fig. 2), 32 patients underwent transanal endoscopic microsurgery (TEM) resection, 40 patients underwent radical surgical resection (Fig. 3) and 12 patients underwent palliative surgical resection (Fig. 4). Distant metastasis occurred in 12 cases.

Of the 195 CR-NETs, 49 cases had lymph node metastasis, with an overall metastatic rate of 25.13% (49/195). Clinicopathological features of lymph node metastasis in CR-NETs are shown in Table 1. The study showed that the differences in lymph node metastasis rate were statistically significance in age (*p* < 0.001), tumour size (*p* < 0.001), tumour location (*p* < 0.001), lymphovascular invasion (*p* < 0.001), muscularis propria invasion (*p* < 0.001), Ki 67 index (*p* < 0.001) and CgA (*p* < 0.001). Multivariate analysis (Table 2) suggested that tumour size ≥ 15 mm (OR: 30.517, 95% CI: 1.250 ~ 744.996, *p* = 0.036) and lymphovascular invasion (OR: 42.796, 95% CI: 2.882 ~ 635.571, *p* = 0.006) were independent risk factors for lymph node metastasis.

**Table 1:** Clinicopathological features of lymph node metastasis in colorectal neuroendocrine tumours

Factors	Patients (*n* = 195)	Lymph node metastasis (*n* = 49)	*p*
Gender, *n* (%)			0.866
Male Female	120 75	31 (25.8%) 18 (24.0%)	
Age, years, *n* (%)			< 0.001
Median (range) < 56 ≥ 56	56 (20–76) 88 107	11 (12.5%) 38 (35.5%)	
Tumor size, mm, *n* (%)			< 0.001
< 15 ≥ 15	141 54	2 (1.40%) 47 (87.0%)	
Tumor location, *n* (%)			< 0.001
Rectum Colon	164 31	23 (14.0%) 26 (83.9%)	
Lymphovascular invasion, *n* (%)			< 0.001
Negative Positive	154 41	14 (9.10%) 35 (85.37%)	
Muscularis propria invasion, *n* (%)			< 0.001
Negative Positive	145 50	3 (2.1%) 46(92.0%)	
Ki 67 index, *n* (%)			< 0.001
≤ 20% > 20%	157 38	13 (8.3%) 36 (94.7%)	
CgA, *n* (%)			< 0.001
Negative Positive	135 60	10 (7.40%) 39 (65.0%)	
Syn, *n* (%)			0.642
Negative Positive	6 189	2 (33.3%) 47 (24.9%)

**Table 2:** Multivariate analysis of factors for lymph node metastasis

Factors	OR	95% CI	*p*
Age, years			0.883
< 56 ≥ 56	1 1.156	Reference 0.168 ~ 7.948	
Tumor size, mm			0.032
< 15 ≥ 15	1 34.295	Reference 1.354 ~ 868.933	
Tumor location			0.856
Rectum Colon	1 0.788	Reference 0.059 ~ 10.434	
Lymphovascular invasion			0.011
Negative Positive	1 24.994	Reference 2.121 ~ 294.510	
Muscularis propria invasion			0.072
Negative Positive	1 17.856	Reference 0.769 ~ 414.536	
Ki 67 index			0.767
≤ 20% > 20%	1 0.642	Reference 0.034 ~ 12.134	
CgA			0.066
Negative Positive	1 6.236	Reference 0.886 ~ 43.917	

*OR* odds ratio, *95% CI* 95% confidence interval

**Fig. 5:**
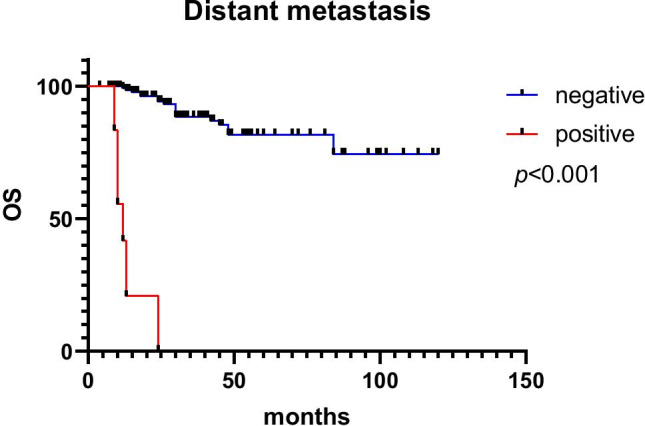
The survival curves according to distant metastasis

**Fig. 6:**
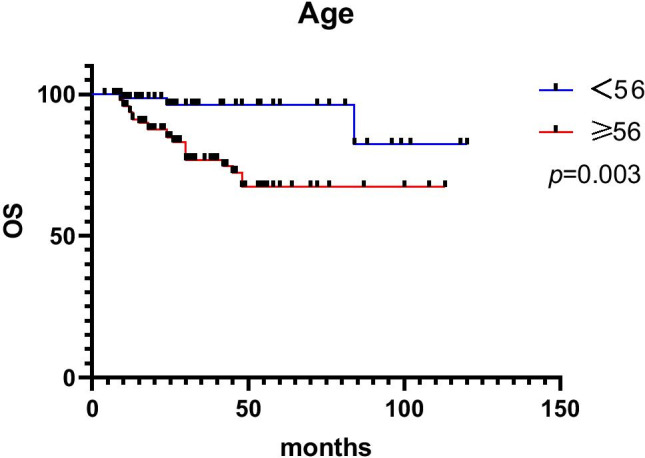
The survival curves according to age

**Fig. 7:**
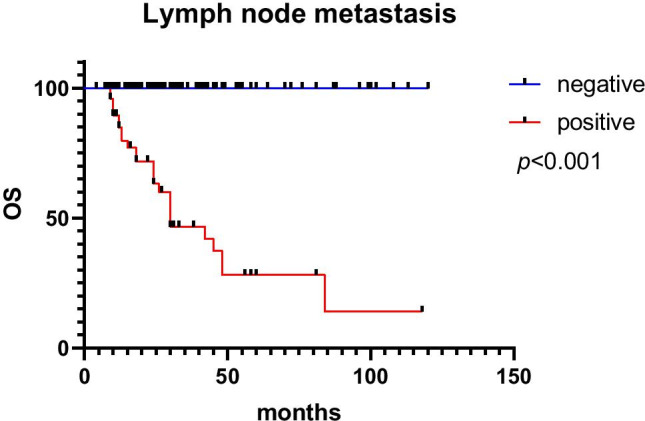
The survival curves according to lymph node metastasis

Of the 195 cases, 21 patients were lost to follow-up, 174 patients were included in the follow-up process and 25 patients died of tumour-related causes. The follow-up time ranged from 6 to 118 months, with a median follow-up time of 28 months, and local recurrence occurred in 2 cases of ESD-resected lesions. Patient age (*p* = 0.009), tumour size (*p* = 0.009), tumour location (*p* < 0.001), lymphovascular invasion (*p* = 0.022), muscularis propria invasion (*p* < 0.001), Ki-67 index (*p* < 0.001), CgA (*p* = 0.006), lymph node metastasis (*p* = 0.016) and distant metastasis (*p* < 0.001) were all associated with prognosis in univariable analyses. Age ≥ 56 (HR: 7.434, 95% CI: 1.334 ~ 41.443, *p* = 0.022) and distant metastasis (HR: 24.487, 95% CI: 5.357 ~ 111.940, *p* < 0.001) were independent prognostic factors in multivariable analyses. Univariable and multivariable Cox proportional hazard regression analyses of OS are shown in Table 3. The survival curves according to distant metastasis, age and lymph node metastasis are shown in Figs. 5, 6 and 7. The 1-year and 5-year survival rates without distant metastasis were 99.3% and 94.3%, respectively. The 1-year and 5-year survival rates with distant metastasis were 41.7% and 0, respectively. The 1-year and 5-year survival rates were 98.5% and 82.4% for those under 56 years of age, 95.8% and 67.4% for those ≥ 56 years of age. The 1-year and 5-year survival rates without lymph node metastasis were 100% and 100%, respectively. The 1-year and 5-year survival rates with lymph node metastasis were 84.7% and 28%, respectively.

**Table 3:** Univariable and multivariable Cox proportional hazard regression analyses of OS

Factors	Univariable			Multivariable		
	HR	95% CI	*p*	HR	95% CI	*p*
Gender						
Male	1	Reference				
Female	0.994	0.439 ~ 2.251	0.988			
Age, years						
< 56	1	Reference		1	Reference	
≥ 56	5.074	1.511 ~ 17.036	0.009	7.434	1.334 ~ 41.443	0.022
Tumor size, mm						
< 15	1	Reference		1	Reference	
≥ 15	347.984	4.347 ~ 27,855.655	0.009	30,372.9	0.00 ~ 1.029E + 74	0.899
Tumor location						
Rectum	1	Reference		1	Reference	
Colon	10.296	4.482 ~ 23.651	< 0.001	1.211	0.455 ~ 3.221	0.702
Lymphovascular invasion						
Negative	1	Reference		1	Reference	
Positive	1.149	1.020 ~ 1.294	0.022	2.053	0.430 ~ 9.800	0.367
Muscularis propria invasion						
Negative	1	Reference		1	Reference	
Positive	79.614	10.738 ~ 590.269	< 0.001	0.343	0.016 ~ 7.344	0.493
Ki 67 index						
≤ 20%	1	Reference		1	Reference	
> 20%	53.017	12.376 ~ 227.117	< 0.001	1.798	0.212 ~ 15.264	0.591
CgA						
Negative	1	Reference		1	Reference	
Positive	224.159	4.624 ~ 10,867.568	0.006	12,536.2	0.000 ~ 1.031E + 74	0.909
Syn						
Negative	1	Reference				
Positive	22.104	0.010 ~ 48,428.080	0.43			
Lymph node metastasis						
Negative	1	Reference		1	Reference	
Positive	578.679	3.338 ~ 100,330.303	0.016	10,529	0.000 ~ 2.823E + 70	0.906
Distant metastasis						
Negative	1	Reference		1	Reference	
Positive	54.618	18.032 ~ 165.430	< 0.001	24.487	5.357 ~ 111.940	< 0.001

*OS* overall survival, *HR* hazard ratio, *95% CI* 95% confidence interval

## Discussion

This study examined the risk factors for lymph node metastasis of CR-NETS, particularly the relationship between tumour size and lymph node metastasis, and derived a new cut-off value (tumour size = 15 mm). Furthermore, it is confirmed that in CR-NETs, size ≥ 15 mm and lymphovascular invasion were independent risk factors for lymph node metastasis, suggesting that these patients should be highly suspected of having lymph node metastasis and should consider the condition comprehensively and carefully select the surgical plan. Meanwhile, age ≥ 56 and distant metastasis were independent prognostic factors.

The NCCN consensus suggests that the size of the primary tumour should be taken as the main criteria for the selection of treatment and monitoring strategies [14]. Endoscopic resection or local resection is recommended for lesions less than 1 cm, and surgical resection is recommended for lesions greater than 2 cm. CR-NETs in between have no clear treatment recommendations. At present, the treatment strategy for tumours between 10 and 19 mm is still controversial [6, 9–11]. Some people recommend radical surgical resection, while others recommend endoscopic resection or local resection; the key factor is whether there is lymph node involvement.

Many past studies have shown a close relationship between tumour size and the risk of metastasis. The reported rate of lymph node metastasis is 1–7% when tumours are smaller than 10 mm [6, 18, 23]. Konishi et al. found that R-NETs with a diameter of 11–20 mm had a higher metastatic potential, with a lymph node metastasis rate of 40% [6]. Soga showed that the metastatic rate for 11–20-mm tumours was 30% [18]. But no exact value was given for grouping tumour sizes. In our study, when 15 mm is the cut-off value, the sensitivity, specificity and AUC of lymph node metastasis were 95.9%, 95.2% and 0.986, respectively. Perhaps we can distinguish tumour size by 15 mm. In our study, when tumour ≥ 15 mm, the lymph node metastasis rate was 93.0%, much higher than 5.9% in the < 15-mm group. Moreover, tumour size ≥ 15 mm was an independent risk factor for lymph node metastasis. For tumours ≥ 15 mm, a higher lymph node metastasis rate should be considered, and the surgical plan should be carefully selected. The research results of Park are consistent with ours [24].

Lymphovascular invasion was an independent risk factor for lymph node metastasis. Some studies were consistent with our conclusions. Lymphovascular invasion should be treated seriously in patients with local resection. In Kang’s study [25], he pointed out that the incidence of lymphatic vascular invasion of small rectal NET was 21.8%, and its occurrence was related to tumour size, and greater than 5 mm was a risk factor for lymphatic vascular invasion. In our study, among 111 cases of endoscopic resection, 6 cases had lymphatic vascular infiltration, and one of them underwent additional surgery without lymph node metastasis. The rest were followed up, and no recurrence or metastasis was found at present, with the longest follow-up period of 6 years. For small rectal NET with lymphatic vascular invasion which resected by endoscopy, Kang et al. recommends a longer follow-up [25].

In our study, age ≥ 56 and distant metastasis were independent prognostic factors. For older patients, it may be due to poor physical fitness, not being able to tolerate the side effects of surgery, drugs and others. Once the tumour has distant metastasis, the patient’s OS is significantly shortened and the prognosis is poor. Even if the primary tumour is removed, the treatment for metastasis will increase the burden on the body; coupled with the double burden of the tumour, the survival will be significantly shortened. At the same time, tumour size, location, lymphovascular invasion, muscularis propria invasion, Ki 67 index, CgA and lymph node metastasis were important prognostic factors. Yu et al. study confirmed that tumour size was an independent prognostic factor [26]. Kim et al. work suggested that CgA expression was an independent predictor of prognosis [27]. Wu et al. study confirmed that age, tumour location, lymph node status and positive level of CgA were independent risk factors affecting prognosis [16]. Therefore, the prognosis of colorectal neuroendocrine tumour cannot be determined by any one factor and needs to be considered comprehensively, especially the poor prognosis with advanced age and distant metastasis.

In this study, 164 NETs occurred in the rectum and 31 in the colon, and most of the tumours in the colon were poorly differentiated. Colonic NETs are a rare malignancy, with an incidence rate of 1 to 2 per million, accounting for less than 1% of all colonic malignancies [28]. With the use of colonoscopy, an increasing number of colorectal tumours have been identified, but well-differentiated colonic NETs are rare. Here, the rate of lymph node metastasis for colonic NET was 83.9%, which was significantly higher than the 14.0% rate in the rectum, and the prognosis was poor. So why does this happen? Is it because colonoscopy is insensitive to lesions in the colon? Or is it due to the rapid development of colonic lesions, short window period and high degree of malignancy? These problems need to be researched in future studies.

We note that the predictors of lymph node metastasis in colorectal neuroendocrine tumours and adenocarcinoma are not identical. Brodsky et al. study confirmed that tumour size was not significant a predictive feature for lymph node metastasis in early rectal cancer [29]. For early colorectal adenocarcinoma, the depth of invasion and tumour budding should be considered more. In Bosch et al. research, submucosal invasion ≥ 1 mm and budding were the strongest independent predictors of lymph node metastasis in early colorectal cancer [30].

Of course, there are some limitations to our study. First, this is a single-centre retrospective study, and there may be bias in the selection of cases. Second, the sample size was not large enough. Third, lymph node evaluation should mainly be based on pathological evaluation after radical resection. However, for local resection cases, we cannot conduct a pathological evaluation but can only conduct imaging evaluation, which may have some errors. Therefore, a large prospective randomized controlled trial is needed to investigate the selection of treatment options for CR-NETs.

In conclusion, when the size of a CR-NET is ≥ 15 mm, the risk of lymph node metastasis is higher, and it is recommended to choose the surgical method carefully. Tumour size and lymphovascular invasion were independent risk factors for lymph node metastasis. Meanwhile, age ≥ 56 and distant metastasis were independent prognostic factors.
